# Vitamin D3 supplementation and treatment outcomes in patients with depression (D3-vit-dep)

**DOI:** 10.1186/s13104-019-4218-z

**Published:** 2019-04-03

**Authors:** Jens Peter Hansen, Manan Pareek, Allan Hvolby, Anne Schmedes, Tomas Toft, Erik Dahl, Connie Thurøe Nielsen

**Affiliations:** 1 Mental Health Services in Esbjerg, Region of Southern Denmark, Gl. Vardevej 101, DK-6715 Esbjerg N, Denmark; 2 The Department of Regional Health Research, University of Southern Denmark, Winsloewparken 19, DK-5000 Odense C, Denmark; 3 Cardiology Section, Department of Internal Medicine, Holbaek Hospital, Smedelundsgade 60, DK-4300 Holbaek, Denmark; 4 Clinical Chemical Laboratory, Lillebaelt Hospital, Beriderbakken 4, DK-7100 Vejle, Denmark; 5 Mental health Services in Odense, Region of Southern Denmark, J.B. Winsloewsvej, DK-5000 Odense C, Denmark; 6 Mental Health Services in Svendborg, Region of Southern Denmark, Baagoeesvej Alle 25, DK-5700 Svendborg, Denmark; 7 Mental Health Services in Vejle, Region of Southern Denmark, Nordbanen 5, DK-7100 Vejle, Denmark

**Keywords:** Depression, Vitamin D deficiency, Randomised controlled trial, Drug therapy, Double-blind method

## Abstract

**Objective:**

To examine whether vitamin D supplementation in patients with depression would result in a reduction in Hamilton D-17 depression score (primary outcome) at 3 and 6 months compared to controls and to explore the correlations between serum vitamin D and symptoms of depression, wellbeing, systolic blood pressure, and waist circumference. In this outpatient multicentre study conducted between 2010 and 2013, patients, 18–65 years old, diagnosed with mild to severe depression were randomly assigned to receive D supplementation 70 micrograms daily or placebo on top of standard treatment. Participants, care givers and those assessing the outcomes were blinded to group assignment.

**Results:**

At baseline, 23 patients had a normal 25(OH)D level, 22 had insufficiency (< 25 nmol/L), and 17 had deficiency (25–50 nmol/L). No significant reduction in depression was seen after vitamin D supplementation compared to placebo at Hamilton (18.4–18.0; p = 0.73 at 12 weeks). Vitamin D supplementation did not provide a reduction in symptom score among patients with depression.

*Trial registration* The trial was registered in the National Board of Health (EudraCT: 2011-002585-20) and in ClinicalTrials.Gov (NCT01390662).

**Electronic supplementary material:**

The online version of this article (10.1186/s13104-019-4218-z) contains supplementary material, which is available to authorized users.

## Introduction

Depression causes important health problems and frequently co-exists with other debilitating chronic conditions [1]. Estimates show that in the European population, the 12-month risk of depression is 6.9%, leading to massive health-related and economic consequences [2].

Guidelines recommend psychotherapy and selective serotonin-reuptake inhibitors or serotonin norepinephrine reuptake inhibitors for patients with this condition). However, approximately 50% of patients with MDD do not respond to first-line antidepressant therapy [3], and the proportion of patients achieving a response decreases to approximately 30% with second-line treatment [4].

Schneider and co-authors have suggested that depressive episodes might be correlated with low levels of vitamin D [5]. Indeed, vitamin D receptors are widespread in the human brain [6], and it has been proposed that low vitamin D status might be involved in the pathogenesis of depression [6, 7]. Furthermore, epidemiological studies show that vitamin D deficiency is associated with an 8–14% increase in the risk of depression [8–11].

Among overweight patients, vitamin D 70–140 μg daily seemed to ameliorate symptoms of depression compared with placebo [12]. However, subsequent meta-analyses did not support this finding [13–15]. Most studies are conducted in patients with low vitamin D levels and without depression. Thus, more studies are needed in patients with depression and concomitant low vitamin D levels [13].

Prior evidence indicates that vitamin D supplementation might have potential benefits as an add-on treatment among patients with depression, particularly during the winter period when levels are low. However, no studies have explored the use of vitamin-D as add-on in regular depression praxis.

Therefore, the aim of this study was to detect whether vitamin D add-on treatment in patients with depression would result in a reduction in depression score at 3 and 6 months compared with controls, and furthermore, to explore the correlations between serum 25(OH)D and symptoms of depression, wellbeing, systolic blood pressure, and waist circumference.

## Main text

### Methods

The study was a randomised, multicentre, double-blind, placebo-controlled trial including patients fulfilling the criteria for a depressive episode according to the International Classification of Diseases (ICD-10) (F32.X) [16].

#### Study population

Participants, consecutively admitted to one of three mood disorder clinics in the Region of Southern Denmark, Esbjerg, Odense and Svendborg, were screened for eligibility in the winter months from November 2010 to March 2014. Patients were eligible if they were suffering from mild to severe depression, 18–65 years old, and had signed a written informed consent form. Exclusion criteria were bipolar affective disorder, any form of schizophrenia, tuberculosis, sarcoidosis, pregnancy, intake of more than 10 μg vitamin D daily, or known allergy/intolerance to the content of the capsules. Women who were in potential of childbearing were excluded if they did not utilize effective contraception. Thus a negative human chorionic gonadotropin (HCG) pregnancy test was required. Patients were excluded if they at baseline had: serum 25(OH)D < 10 nmol/L or > 100 nmol/L, serum calcium (ionised) > 1.40 mmol/L, estimated glomerular filtration rate (eGFR) < 60 mL/min/1.73 m^2^, serum phosphate < 1.50 mmol/L (females) or < 1.60 mmol/L (males aged 18–49 years) or < 1.35 mmol/L (males > 49 years), or serum parathyroid hormone (PTH) > 9.2 pmol/L.

#### Interventions

Participants were randomly assigned to receive either vitamin D [70 μg vitamin D3 (2800 IU)] or placebo. Placebo capsules contained lactose. Participants were provided with 12 weeks of study medication. Both groups received treatment as usual including psychiatric examination with diagnostic interview, cognitive behaviour therapy, psychoeducation and psychotropic medication according to national guidelines.

#### Randomisation and blinding

The participants were randomised into two groups (intervention or control) using blocks of four. The randomisation procedure was computer-generated and conducted in a labelling procedure concealed for staff and researchers having implications for the trial throughout the study. The capsules were produced in Denmark, identical in size, smell, and taste.

The primary outcome was the sum of the Hamilton Rating Scale for Depression (17-items) (HAM-D17).

The secondary outcomes were the sum of the of the validated self-reported well-being and Major Depression Inventory (MDI), [17], World Health Organization-Five Well-Being Index (WHO-5).

#### Assessments

Depression level and mental health was assessed and diagnosed by an experienced psychiatrist.

The primary outcome was assessed using HAM-D17 [18, 19]. The secondary outcomes were assessed using WHO-5 [26] and MDI all in Danish versions.

All outcomes were assessed at baseline and at 3 and 6 months. Additionally weight, waist circumference, blood pressure and 25(OH)D were assessed at 3 and 6 months. Socio-demographic factors were assessed at baseline. Known side effects of vitamin D supplementation, and severe adverse events, use of dietary supplements and a full medication list were recorded at baseline and follow-up. The assessments were conducted by trained specialist nurses.

Treatment adherence was assessed by the counting the number of capsules returned by the patients at 12 weeks.

25(OH)D, C-reactive protein (CRP), phosphate, ionised calcium, and PTH were measured. PTH analyses were conducted on Immulite 2000 (Siemens). 25(OH)D was measured by high performance liquid chromatography followed by tandem mass spectrometry. The method quantifies 25-hydroxyvitamin D3.

#### Statistical analyses

Differences in baseline characteristics between the intervention and control groups were investigated using Fisher’s exact test for categorical variables and Student’s t-test for continuous variables. Differences between the two groups for each of the primary and secondary outcomes were also tested using multivariable regression analyses. Correlations between 25(OH)D and HAM-D17, WHO-5, systolic blood pressure, and waist circumference were analysed using mixed model analysis. The analyses were conducted according to intention-to-treat principles.

#### Power calculation

With a hypothesised mean decrease of 3 points (standard deviation: 4.5) in the HAM-D17 score for controls versus placebo for 12 weeks, we required 80 patients in each group to reject the null-hypothesis of no between-group difference in HAM-D17, at an alpha of 5%, with a power of 80%.

### Results

#### Participant flow and baseline characteristics

**Fig. 1:**
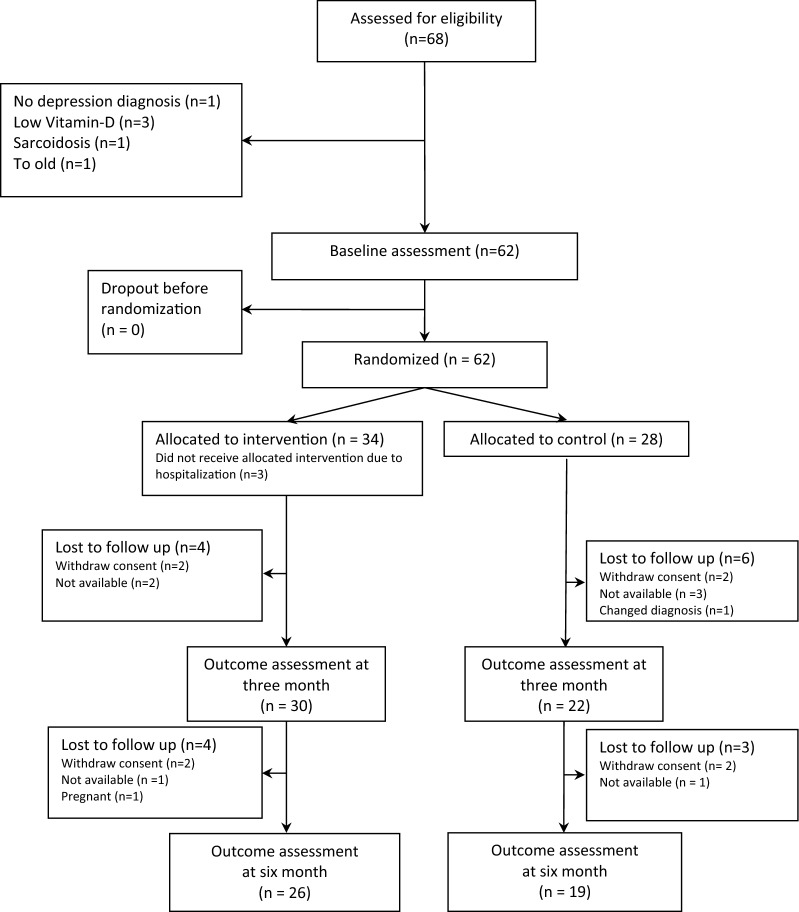
Flow chart

A participant flowchart is provided in Fig. 1. All 68 eligible patients were invited to participate in the study. Before randomisation, 6 were excluded due to abnormal blood samples, mainly abnormal vitamin D and PTH levels. Sixty-two patients were included in the trial.

#### Demographic data

As shown in Table 1, 23 patients had a normal 25(OH)D level; 22 had levels indicating vitamin D insufficiency and in 17 deficiency. Of the 62 patients, 34 received vitamin D, and 28 received placebo. Forty-four individuals completed the study, and outcome data were collected from September 2011 to June 2014. Most of the patients were included during the winter period.

**Table 1:** Demographic and clinical characteristics of participants at baseline

	Intervention group (n = 28)	Control group (n = 34)	Total (n = 62)
Female, n (%)	21 (75.0)	23 (67.7)	47 (69.1)
Age (years), mean (SD)	39.6 (13.5)	38.7 (11.4)	39.1 (12.3)
Normal 25(OH)D (≥ 50 nmol/L), n (%)	10 (35.6)	13 (38.2)	23 (35.5)
Insufficient 25(OH)D (25–50 nmol/L), n (%)	11 (39.3)	11 (32.4)	22 (35.5)
Deficient 25(OH)D (< 25 nmol/L), n (%)	7 (25.0)	10 (29.4)	17 (27.4)
SSRI, n (%)*	17 (60.7)	12 (35.3)	29 (46.8)
SNRI, n (%)	12 (42.9)	18 (52.9)	30 (48.4)
Depression (HAM-D17), mean (SD)	18 (6)	18 (6)	18 (6)

*SSRI* selective serotonin reuptake inhibitor, *SNRI* serotonin–norepinephrine reuptake inhibitors

* Significantly different between the two groups (p = 0.046)

Dropout-analyses revealed a significantly lower age among dropouts than among completers (p = 0.01), with a mean age of 41.8 years (SD = 12.7) for completers and 33.1 (SD = 9.3) for dropouts.

#### Primary outcome

Baseline mean values for HAM-D17 did not significantly differ between the intervention group 18.2 (SD = 8.8) and the control group 18.0 (SD = 5.7). There was a decrease in HAM-D17 from baseline to study completion in both groups (p < 0.001) (Table 2). However, there was no significant between-group difference in the decrease of HAM-D17 over time at 6 months (intervention group, mean decrease − 9.58 (SD = 6.2); control group, mean decrease: − 7.1 (SD = 6.2); p = 0.17).

**Table 2:** Clinical outcomes of for the patients in the intervention group and in the control group

	Intervention group (n = 28)			Control group (n = 34)			p values	
	Baseline	12 weeks	24 weeks	Baseline	12 weeks	24 weeks	12 weeks	24 weeks
Hamilton	18.4 (5.73)	10.6 (5.40)	9.26 (6.32)	18.0 (6.01)	9.50 (5.48)	9.59 (7.82)	0.73	0.17
MDI	33.4 (10.7)	21.8 (10.5)	16.4 (12.0)	33.8 (7.77)	20.4 (10.5)	19.5 (11.6)	0.83	0.57
WHO-5	23.8 (15.3)	37.0 (25.3)	50.5 (29.0)	24.0 (17.2)	39.5 (21.2)	47.2 (22.4)	0.89	0.49
Vitamin D	43.2 (24.6)	94.5 (30.0)	97.9 (25.0)	44.3 (24.1)	44.4 (25.0)	52.0 (33.5)		

Values are mean (SD) unless stated otherwise

*Hamilton* Hamilton Rating Scale for depression (HRSD-17), *MDI* major depression inventory, *WHO5* WHO5 Well-being Index, *Vitamin D* 25(OH)D level

The mean (SD) decrease in the intervention were: − 9.58 (6.2); the mean (SD) decrease in the control group were: − 7.1 (6.2); (p = 0.17). The decrease in depression scores in both groups were significantly (p < 0.001)

Subgroup analysis for patients having a vitamin D level below 50 nmol/L at baseline revealed no significant differences in depression scores (see Additional file 1: Table S1) at endpoint. The analysis did not reveal any interaction with centre and no significant effect of month of inclusion. However, we did not find any difference between centres.

#### Secondary outcomes

No association between 25(OH)D levels and HAM-D17 (p = 0.89), WHO-5 (p = 0.77), or waist circumference (p = 0.23) was found (Additional file 2: Table S2). However, systolic blood pressure was negatively associated with 25(OH)D (p = 0.03) (see Additional file 2: Table S2). We further found no significant differences in the effect on systolic blood pressure 3 months (p = 0.15) or at 6 months (p = 0.08).

#### Side effects and adverse events

There were no significant between-group differences in known side effects or other adverse events (see Additional file 3: Table S3). None of the other adverse events were related to vitamin D. One patient in the control group and one patient in the intervention group had a PTH level above the reference interval (PTH > 9.2).

### Discussion

In this randomised double-blind study of vitamin D supplementation in patients with depression, we found no significant reductions in depression score at 3 and 6 months.

The design of the study including block randomisation was appropriate when investigating effect of vitamin D during winter time. Additionally, the study was conducted using a double-blind randomised design.

However, the design, although appropriate, did not give positive results due to low power, although there was a significant decrease in depressions scores in the intervention group (p < 0.001). The similar high response rate in the control group might come from a significant effect from the treatment as usual. The effect of the intervention might be too low compared to an effective standard treatment.

The robust temporal changes in primary outcome in both groups are likely explained by an effective standard treatment regimen, including psychotherapy and antidepressants. Thus, the additive effective of vitamin D is probably negligible. However the study might have given significant results if conducted with full power and including exclusively participants with low vitamin D.

Our results are similar to those of three meta-analyses showing no significant benefit of vitamin D on depression [13–15]. However, one of the meta-analysis indicated positive results in studies without biological flaws [14]. The design in the present study is different since all the participants in this study had a diagnosis of depression, but the population size was too small to determine whether vitamin D is useful in a population receiving effective standard treatment for depression.

## Limitations

The study has several limitations. We did not reach the estimated sample size, and the study was grossly underpowered. We included participants from three centres. The staff had the task to inform the patients and give the names to the responsible researcher. However fewer patients than expected were interested in participating in the study. We might include more centres. However the founding did not give us the opportunity to include those. Moreover, only a minority of participants were included from two of the centres. The latter possibly gave inefficient block randomisation with uneven group sizes. We included patients with a normal vitamin D level. This could minimise the chance of detecting an effect of vitamin D supplementation. Sub-analysis using a vitamin D cut-off level of 50 nmol/L did not alter results.

## Additional files

**Additional file 1: Table S1.** Clinical outcomes of for patients having a vitamin D below 50 nmol/L at baseline. Table of Hamilton, MDI, WHO-5 and Vitamin D results at baseline, 12 weeks and 24 weeks including p-values.

**Additional file 2: Table S2.** Correlations between serum 25(OH)D and selected outcomes. Correlations between Vitamin D and Hamilton, WHO-5, MDI, Systolic blood pressure and Waist circumference including p-values.

**Additional file 3: Table S3.** Side effects and related measures for both groups. Table of percentage of patients having side effects (nausea, constipation, kidney stone og other side effects and maximum values of selected outcomes (calcium, phosphate and PTH). The table consists results from the intervention and the control group.

## Abbreviations

BDI: Beck Depression Index
CRP: C-reactive protein
eGFR: estimated glomerular filtration rate
ICD-10: international classification of diseases
HAM-D17: Hamilton Rating Scale for Depression (17-items)
HCG: human chorionic gonadotropin
MDD: major depressive disorder
PTH: para thyroid hormone
WHO-5: World Health Organization-Five Well-Being Index
