# Supplementary material for: Vitamin D3 supplementation and treatment outcomes in patients with depression (D3-vit-dep)
Source: BMC Res Notes. 2019 Apr 3;12:203. doi: 10.1186/s13104-019-4218-z (PMC6446320; doi:10.1186/s13104-019-4218-z)
Supplement: Supplementary file 1 — Additional file 1: Table S1. Clinical outcomes of for patients having a vitamin D below 50 nmol/L at baseline. Table of Hamilton, MDI, WHO-5 and Vitamin D results at baseline, 12 weeks and 24 weeks including p-values. [file 13104_2019_4218_MOESM1_ESM.docx]

| Table S1: Clinical outcomes of for patients having a vitamin D below 50 nmol/l at baseline | | | | | | | | | | |  | |
| --- | --- | --- | --- | --- | --- | --- | --- | --- | --- | --- | --- | --- |
|  | Intervention group (n=19) | | | Control group (n=25) | | | | |  | | | |
|  | Baseline | 12 weeks | 24 weeks |  | Baseline | 12 weeks | 24 weeks | | | P value 12 weeks | | P value 24 weeks |
| Hamilton | 19.1 (5.77) | 10.4 (5.18) | 9.57 (6.85) |  | 17.8 (5.48) | 8.89 (6.13) | 9.47 (8.19) | | | 0.67 | | 0.82 |
| MDI | 33.7 (11.5) | 22.8 (9.73) | 15.4 (11.2) |  | 32.3 (7.87) | 19.3 (10.3) | 20.7 (11.8) | | | 0.20 | | 0.34 |
| WHO-5 | 23.8 (16.9) | 36.3 (24.7) | 50.5 (31.7) |  | 23.2 (13.3) | 41.3 (23.0) | 47.2 (23.4) | | | 0.25 | | 0.76 |
| Vitamin D | 28.4 (10.2) | 87.7 (26.3) | 96.5 (25.6) |  | 30.7 (11.3) | 36.9 (19.2) | 41.8 (21.3) | | |  | |  |
| Values are mean (SD) unless P value | | | | | | | |  | | | | |
| Hamilton = Hamilton Rating Scale for depression (HRSD-17) | | | | | | | | | | | | |
| MDI = Major depression Inventory | | | | | | | | | | | | |
| WHO5 = WHO5 Well-being Index  Vitamin D = 25(OH)D level | | | | | | | | | | | | |
