# Supplementary material for: Vitamin D3 supplementation and treatment outcomes in patients with depression (D3-vit-dep)
Source: BMC Res Notes. 2019 Apr 3;12:203. doi: 10.1186/s13104-019-4218-z (PMC6446320; doi:10.1186/s13104-019-4218-z)
Supplement: Supplementary file 2 — Additional file 2: Table S2. Correlations between serum 25(OH)D and selected outcomes. Correlations between Vitamin D and Hamilton, WHO-5, MDI, Systolic blood pressure and Waist circumference including p-values. [file 13104_2019_4218_MOESM2_ESM.docx]

| Table S2: Correlations between serum 25(OH)D and selected outcomes | | | | | |
| --- | --- | --- | --- | --- | --- |
|  | Beta Coef. | [95% Conf. Interval] | | | P value |
| Hamilton | 0.00 | -0.03 | 0.28 |  | 0.89 |
| WHO-5 | -0.02 | 0.13 | 0.09 |  | 0.77 |
| MDI | 0.10 | -0.04 | 0.06 |  | 0.67 |
| Systolic BP | -0.07 | - 0.14 | -0.01 |  | 0.03 |
| Waist -circumference | 0.02 | -0.01 | 0.05 |  | 0.23 |
| Hamilton = Hamilton Rating Scale for depression (HRSD-17)  MDI = Major depression Inventory  WHO5 = WHO5 Well-being Index  BP = Blood pressure | | | | | |
