# Supplementary material for: Vitamin D3 supplementation and treatment outcomes in patients with depression (D3-vit-dep)
Source: BMC Res Notes. 2019 Apr 3;12:203. doi: 10.1186/s13104-019-4218-z (PMC6446320; doi:10.1186/s13104-019-4218-z)
Supplement: Supplementary file 3 — Additional file 3: Table S3. Side effects and related measures for both groups. Table of percentage of patients having side effects (nausea, constipation, kidney stone og other side effects and maximum values of selected outcomes (calcium, phosphate and PTH). The table consists results from the intervention and the control group. [file 13104_2019_4218_MOESM3_ESM.docx]

| Table S3: Side effects and related measures for both groups | | | | |
| --- | --- | --- | --- | --- |
|  | 12 week | | 24 week | |
|  | Intervention  group | Control  group | Intervention  group | Control  group |
| Nausea or vomit (percentage) | 22.7 | 21.7 | 22.2 | 15.8 |
| Constipation (percentage) | 27.3 | 30.4 | 5.6 | 25.0 |
| Kidney stone (percentage) | 0 | 0 | 0 | 0 |
| Other side effects (percentage) | 50.0 | 39.1 | 50.0 | 45.0 |
| Maximum serum calcium (values) | 1.28 | 1.28 | 1.3 | 1.27 |
| Maximum serum phosphate (values) | 1.41 | 1.21 | 1.29 | 1.24) |
| Maximum PTH (values) | 7.4 | 9.7 | 8.2 | 9.9 |
